# Identification of gene variation feature for targeted therapy of non-small cell lung cancer through combined method of DNA and RNA sequencing

**DOI:** 10.1007/s12672-024-00915-3

**Published:** 2024-03-06

**Authors:** Wenguang Pang, Longlong Gong, Wangpan Shi, Hongbo Zheng, Min Ye, Jiarong Chen, Ronggang Li, Xin Zhang, Dong Ren, Zheng Wang

**Affiliations:** 1 The First Affiliated Hospital of Jinan University, Guangzhou, 510630 China; 2 Department of Thoracic Surgery, Jiangmen Central Hospital, Jiangmen, 529030 China; 3 Genecast Biotechnology Co., Ltd., Wuxi, 214104 China; 4 Department of Pathology, University of California San Diego Health System, San Diego, CA 92037 USA; 5 Department of Oncology, Jiangmen Central Hospital, Jiangmen, 529030 China; 6 Department of Pathology, Jiangmen Central Hospital, Jiangmen, 529030 China; 7 Clinical Experimental Center, Jiangmen Key Laboratory of Clinical Biobanks and Translational Research, Jiangmen Central Hospital, Jiangmen, 529030 China; 8 Departments of Pathology, University of California Irvine Medical Center, 101 The City Drive South, Orange, CA 92868 USA; 9 Department of Thoracic Surgery, The 2nd Clinical Medical College of Jinan University, Shenzhen, 518020 China

**Keywords:** Next generation sequencing, Non-small cell lung cancer, Single-nucleotide variations, Copy number variations, Gene fusion

## Abstract

Next generation sequencing (NGS) is typically used to reveal tumor gene variation feature for targeted therapy of various types of human cancers, including non-small cell lung cancer (NSCLC). Here, we report the role and potential applicable value of combining DNA and RNA sequencing in gene variation detection in NSCLC. 386 NSCLC patients with stage II-IV were enrolled and detected using NGS sequencing of DNA and RNA panels that covered all well-documented target driver genes from the Chinese Society of Clinical Oncology (CSCO). The rate of epidermal growth factor receptor (EGFR) single nucleotide variation (SNV)/indel, mesenchymal-epithelial transition factor (MET) copy number variation (CNV) and anaplastic lymphoma kinase (ALK) fusion were 52.1%, 4.1% and 6.0% in the NSCLC cohort. The landscapes of SNV/indel, CNV and gene fusion in the cohort were depicted as well. Meanwhile, we assessed detection efficacy of DNA and RNA sequencing in gene fusion. Detected number and types of gene fusion using the RNA sequencing were better than those using the DNA sequencing. Gene fusion with intergenic region was only detected by DNA sequencing and MET exon 14 skipping (METΔex14) was more easily identified by RNA sequencing. Finally, we investigated clinical correlations of SNV/indel/CNV/fusion with clinicopathologic features in the NSCLC cohort. Taken together, RNA sequencing significantly complements deficiency of DNA sequencing for gene fusion, which cooperatively presents comprehensive and reliable gene variation features and facilitate the identification of potential drug targets for NSCLC patients.

**Supplementary Information:**

The online version contains supplementary material available at 10.1007/s12672-024-00915-3.

## Introduction

Lung cancer remains the leading cause of cancer-related death worldwide, where non-small cell lung cancer (NSCLC) accounts for approximately 85 percent of lung cancer cases [1]. With substantial development and extensive application of targeted drugs against driver mutations, outcome and survival of NSCLC patients have been significantly improved [2, 3]. Notably, the Chinese Society of Clinical Oncology (CSCO) guideline (Version 2022) distributes recent treatment recommendations for NSCLC, including detection and target therapy of single-nucleotide variations (SNV)/insertions/deletions (indel) of epidermal growth factor receptor (EGFR), Kirsten rat sarcoma viral oncogene homolog (KRAS), human epidermal growth factor receptor 2 (HER2) and serine/threonine-protein kinase BRAF genes, copy number variations (CNV) of mesenchymal-epithelial transition factor (MET) and HER2, gene fusions of anaplastic lymphoma kinase (ALK), ROS proto-oncogene 1 (ROS1), rearranged during transfection (RET) and neurotrophin receptor kinase (NTRK), and MET exon 14 skipping (METΔex14). NSCLC patients with driver gene mutations, such as EGFR mutation, presented favorable outcome and survival after treatment of Icotinib, Osimertinib and Gefitinib [4–6]. Alectinib and ceritinib significantly enhanced progression-free survival (PFS) in ALK-rearranged NSCLC patients [7, 8]. Capmatinib showed substantial antitumor activity in advanced NSCLC patients with a METΔex14 or MET amplification, particularly in patients without previous treatment history [9]. Therefore, it is conceivable that accurate and comprehensive investigation of specific gene mutation for NSCLC patients might significantly improve the outcome and prognosis of NSCLC patient through using target-drug treatment.

Next generation sequencing (NGS) technology has been undoubtedly recommended by the National Comprehensive Cancer Network (NCCN) and CSCO guidelines for the detection of high-throughput gene variations, which will guide target therapy for NSCLC patients [10]. Genomic DNA (gDNA) and whole exome sequencing (WES) are currently and typically used for analysis of SNV, indel and CNV in tumor tissue samples in NGS-based target therapy selection, and messenger RNA (mRNA) has been more widely adopted as input template for identification of fusions and gene expression analysis. However, mRNA is rarely used to detect somatic mutations such as SNVs or indels due to variable expression levels due to limited accuracy. 46–49% of pathogenic variants identified by DNA sequencing can only be detected through RNA sequencing analysis, especially all telomerase reverse tranase (TERT) mutations failed to be identified by RNA sequencing [11]. Thus, combination of DNA and RNA sequencing could comprehensively and accurate explore gene variation feature as mutual complement for NSCLC patients.

The present study aimed at developing an inexpensive and scalable panel DNA/RNA-seq method capable of detecting multiple types of clinically actionable mutations with high accuracy in NSCLC. 386 NSCLC patients with stage II-IV were enrolled and detected using NGS sequencing of DNA and RNA panels. The DNA panel (769 genes) and RNA panel (29 genes) covered all well-documented target driver genes from the CSCO guideline for NSCLC. We systematically analyzed gene variation feature and explored gene fusion feature between DNA panel and RNA panel sequencing in the NSCLC cohort. Clinical correlations of these gene variations/fusion with clinicopathological feature have been further elucidated in the NSCLC cohort. Therefore, combination of DNA and RNA panel sequencing will facilitate comprehensive identification of gene variation and potential drug targets so as to improve the outcome and prognosis of every single NSCLC patient according to the specific gene mutation. Meanwhile, fewer tumor samples, more detecting depth and less cost of time and material with DNA and RNA panels provided the foundation for wide clinical application in NSCLC.

## Materials and methods

### Patients and clinical data

386 NSCLC patients with stage II-IV were obtained from Jiangmen Central Hospital and Genecast Clinical Laboratory Center from June 2020 to October 2022. The detailed clinical information was summarized in Table 1. In the cohort, cases with stage II, III and IV occupied 16.3%, 32.9% and 50.8%, respectively. 339 patients (87.8%) were lung adenocarcinoma (LUAD) and 47 patients (12.2%) were lung squamous cell carcinoma (LUSC). The study was reviewed and approved by the Ethics Committee of Jiangmen Central Hospital (2022–119) and all tests were performed according to relevant guidelines.

**Table 1:** Clinical characteristics

Factor	NSCLC (n = 386)
Age (Median, quartiles)	63 (31–87)
Gender (n, %)	
Male	207 (53.6%)
Female	179 (46.4%)
Smoking (n, %)	
Yes	132 (34.2%)
No	212 (54.9%)
NA	42 (10.9%)
Clinical stage (n, %)	
II	63 (16.3%)
III	127 (32.9%)
IV	196 (50.8%)
Tumor subtype (n, %)	
LUAD	339 (87.8%)
LUSC	47 (12.2%)

### DNA sequencing

Detail methods of DNA sequencing was performed as our previous study [12]. DNA panel was 2,189 kb in length, which covered full coding regions or hotspot mutation regions in 769 genes. The genomic DNA from formalin-fixed paraffin-embedded (FFPE) tumor samples was extracted using the MagPure FFPE DNA Kit B (Magen, China), fragmented into DNA pieces of approximately 200 bp using an enzymatic method (5 × FEA Enzyme Mix; Qiagen, CN), subsequently constructed DNA libraries using a VAHTS Universal DNA Library Prep Kit (Vazyme, China). The libraries of genomic DNA were captured with a 769 gene panel (Table S1) and sequenced using a HyperCap Target Enrichment Kit (Roche, Switzerland) and the NovaSeq 6000 system (Illumina, USA) according to the manufacturer’s protocols, respectively.

### RNA sequencing

RNA panel was 126 kb in length, which covered whole transcript sequences of 29 genes. RNA was extracted from FFPE samples using the MagPure FFPE RNA/DNA Kit (Magen, China), and FFPE RNA (50–300 ng) was employed for library construction using mRNA-seq Lib Prep Module for Illumina (ABclonal, China). The RNA panel (29 genes, Table S2) was synthesized by IDT (Integrated DNA Technologies, USA). Hybridization and washing were performed using the xGen Hybridization and Wash Kit and xGen Universal Blockers-TS Mix-96rxn (IDT, USA) according to manufacturer’s instructions. The captured products were sequenced on Illumina NovaSeq 6000.

### Statistical analysis

All statistical analysis was performed using GraphPad Prism 8.4.2 and R4.2.1. Analysis SNV/indel and CNV landscapes was performed using a Complex Heatmaps package (2.12.1). Sequence alignment was performed using Integrative Genomics Viewer (IGV) analysis. The Fisher’s exact test was used to analyze the frequency difference of SNV and gene fusion between the two/three groups. The unpaired *t* test and One Way ANOVA were used to analyze the difference of CNV between two and three groups, respective*ly. p* < 0.05 was considered statistically significant.

## Results

### Potential well-documented target driver genes from the CSCO guideline for NSCLC using NGS

**Fig. 1:**
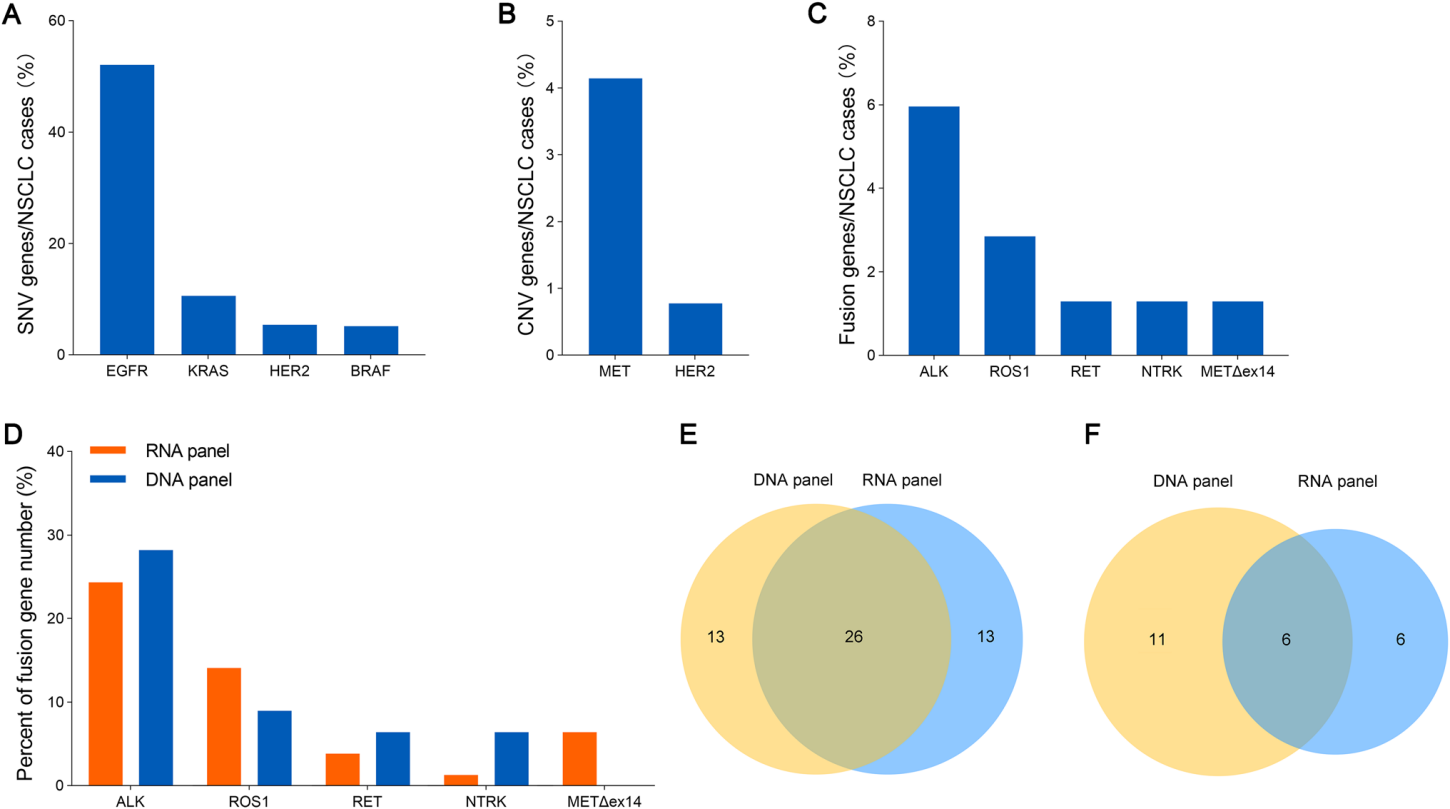
Roles of NGS detection in diagnostic and treatment of NSCLC according to the Chinese Society of Clinical Oncology (CSCO) clinical guidelines. **A** SNV/indel mutations of EGFR, KRAS, HER2 and BRAF in NSCLC cases (n = 386) detected by DNA panel sequencing. **B** CNV mutations of MET and HER2 in NSCLC cases (n = 386) detected by DNA panel sequencing. **C** ALK, ROS1, RET and NTRK fusion and METΔex14 in NSCLC cases (n = 386) detected by DNA and RNA panel sequencing. **D** Percent of ALK, ROS1, RET and NTRK fusion and METΔex14 detected in NSCLC cases by DNA and RNA sequencing, respectively. **E**–**F** Gene fusion number **E** and fusion types **F** of ALK, ROS1, RET, NTRK and METΔex14 detected by DNA or RNA panel sequencing

According to the NSCLC CSCO 2022 guideline, detection of SNV/indel of EGFR, KRAS, HER2 and BRAF genes, CNV of MET and HER2, gene fusions of ALK, ROS1, RET and NTRK, and MET exon 14 skipping (METΔex14) would facilitate clinical application of target drugs for NSCLC patients based on these gene variations. DNA panel sequencing (769 genes) covering all aforementioned driver genes was used for the SNV/indel detection of EGFR, KRAS, HER2 and BRAF genes (Fig. 1A), and CNV of MET and HER2 genes (Fig. 1B). The number of SNV/indel of EGFR was 201 cases (52.1%), and the number of CNV of MET was 16 cases (4.1%) in the NSCLC cohort (Fig. 1A, B). Gene fusions of ALK, ROS1, RET, NTRK, and METΔex14 were detected by sequencing of DNA and RNA panels (Fig. 1C).

Gene fusions were further explored using DNA panel or RNA panel sequencing in the NSCLC cohort. However, fisher's exact test showed that of the DNA and RNA panel sequencing was not significantly different in detection of ALK, ROS1, RET, NTRK, and METΔex14 genes (*p* > 0.05, Fig. 1D). The number of gene fusion containing ALK, ROS1, RET, NTRK or METΔex14 was comparable in the NSCLC cohort (Fig. 1E). 50% (26/52) gene fusion was simultaneously detected by both strategies and the remaining 50% was complementarily found by DNA or RNA panel sequencing (Fig. 1E). Surprisingly, detection rate of gene fusion types with ALK, ROS1, RET, NTRK or METΔex14 by DNA panel sequencing were higher than that using RNA panel (Fig. 1F). These data indicated that DNA sequencing combined with RNA sequencing could more comprehensively and reliably exhibit variations of genes in the NSCLC cohort.

### Gene variation landscapes in the NSCLC cohort with DNA and RNA panel sequencing

**Fig. 2:**
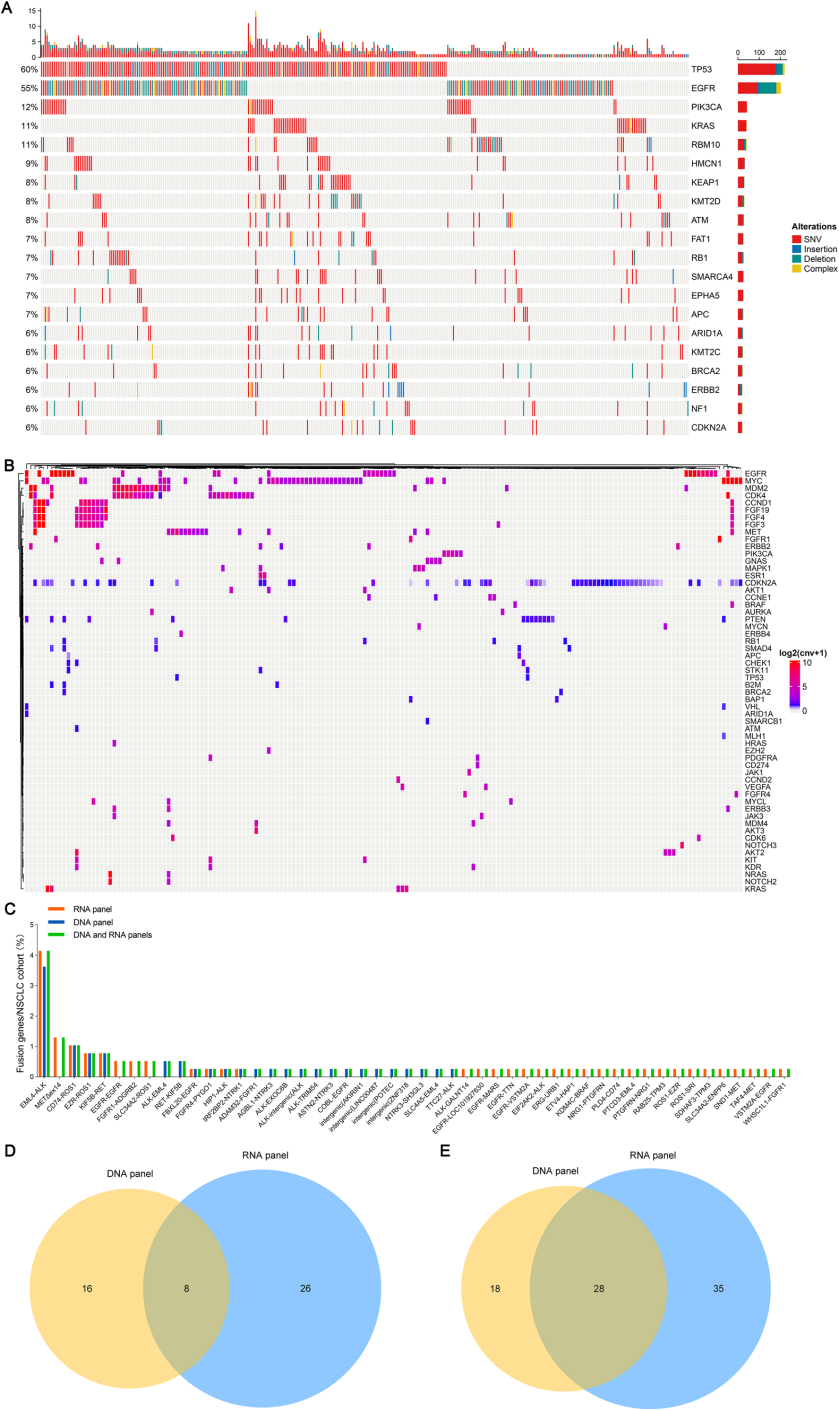
Landscape of SNV/indel, CNV and gene fusion in the NSCLC cohort. **A** SNV/indel landscape of top 20 genes using DNA panel sequencing in NSCLC cases (n = 365). Gene variations contained SNV, insertion, deletion and complex mutations. **B** CNV landscape using DNA panel sequencing in NSCLC cases (n = 172). There were 58 genes with CNV in the cohort. Gene copy number was showed using log2(CNV + 1). **C** Gene fusion using DNA and RNA panels sequencing in NSCLC cases (n = 386). Orange, blue and green presented fusion genes of RNA panel, DNA panel and common detection with RNA and DNA panels, respectively. **D** Venn diagram of the number of fusion genes detected by DNA panel and RNA panel in NSCLC cases (n = 386). **E** Venn diagram of the number of gene fusion types detected by DNA panel and RNA panel in NSCLC cases (n = 386)

We further displayed SNV/indel landscape of top 20 genes using DNA panel sequencing in NSCLC cases, which indicated that the SNV/indel frequencies of TP53 and EGFR were 60% and 55%, respectively (Fig. 2A). CNV landscapes of all genes in the NSCLC cases were showed in Fig. 2B. CNV rates of CDKN2A, myelocytomatosis oncogene (MYC), EGFR and cyclin-dependent kinases 4 (CDK4) genes in the NSCLC cohort were 15.9%, 12.4%, 8.2% and 7.4%, respectively. CDKN2A gene was loss of copy number, and MYC, EGFR and CDK4 genes were gain of copy number. Gene fusion landscapes was showed in Fig. 2C in the NSCLC cohort using the DNA and RNA panel sequencing. Echinoderm microtubule-associated protein-like 4 (EML4)-ALK fusion detected by NGS was most in all gene fusions and the rate was 8.2% in the NSCLC cohort. Although fusion detection rate of genes from the CSCO guideline with DNA and RNA panels was similar (Fig. 1E, F), the number and types of gene fusion detected using the RNA panel sequencing were more than those through the DNA panel sequencing (Fig. 2D, E). Thus, sequencing of the RNA panel greatly complemented deficiency of the DNA panel for gene fusion in the NSCLC cohort, which cooperatively revealed a more comprehensive gene variation feature in NSCLC.

### Detection of rare gene fusion with DNA and RNA panel sequencing

**Fig. 3:**
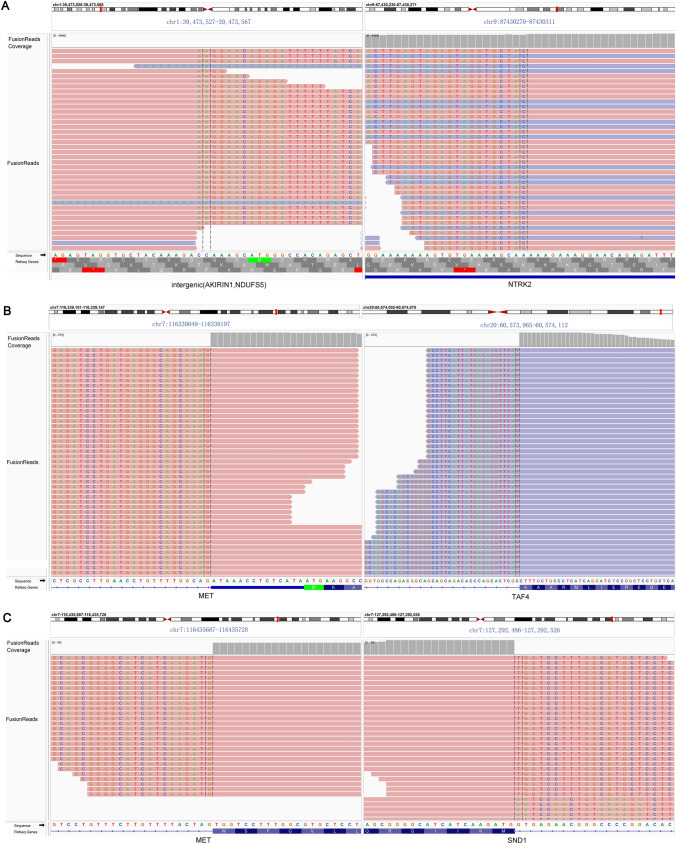
Detection of rare gene fusion with sequencing of the DNA and RNA panels. **A** Intergenic(AKIRIN1,NDUFS5)-NTRK2 fusion was only detected by sequencing of DNA panel. **B** TAF4-MET fusion was only detected by sequencing of RNA panel. **C** SND1-MET fusion was only detected by sequencing of RNA panel

By definition, fusion gene that was detected only once with DNA and/or RNA panels in the NSCLC cohort, was regarded as a rare gene fusion. As shown in Fig. 3A, there were 40 rare fusions identified in the cohort. Among them, 4 gene fusions, including F-box and leucine-rich repeat protein 20 (FBXL20)-EGFR, fibroblast growth factor receptor (FGFR4)-PYGO1, hedgehog-interacting protein 1(HIP1)-ALK and interferon regulatory factor-2-binding protein-2 (IRF2BP2)-NTRK1, were simultaneously discovered using DNA and RNA panel sequencing. 14 (35%) rare fusions were detected by the DNA panel and 22 (55%) rare fusions were detected by the RNA panel. The gene fusion with intergenic region was only detected by DNA panel, including intergenic (AKIRIN1, NDUFS5)-NTRK2, intergenic (ZNF318, ABCC10)-EZR, intergenic (POTEC, ANKRD30B)-PDGFB, intergenic (LINC00487, NRIR)-ALK and ALK-intergenic (ALK, YPEL5) (Fig. 3A). Gene fusion with driver genes such as TAF4-MET and SND1-MET detected by the RNA panel sequencing might provide a target-drug direction (Fig. 3B, C). In addition, the gene variants outside the spectrum of panels were not measured, which needs to be further assessed using whole genetic sequencing or transcriptome sequencing.

### METΔex14 detection using DNA and RNA sequencing

**Fig. 4:**
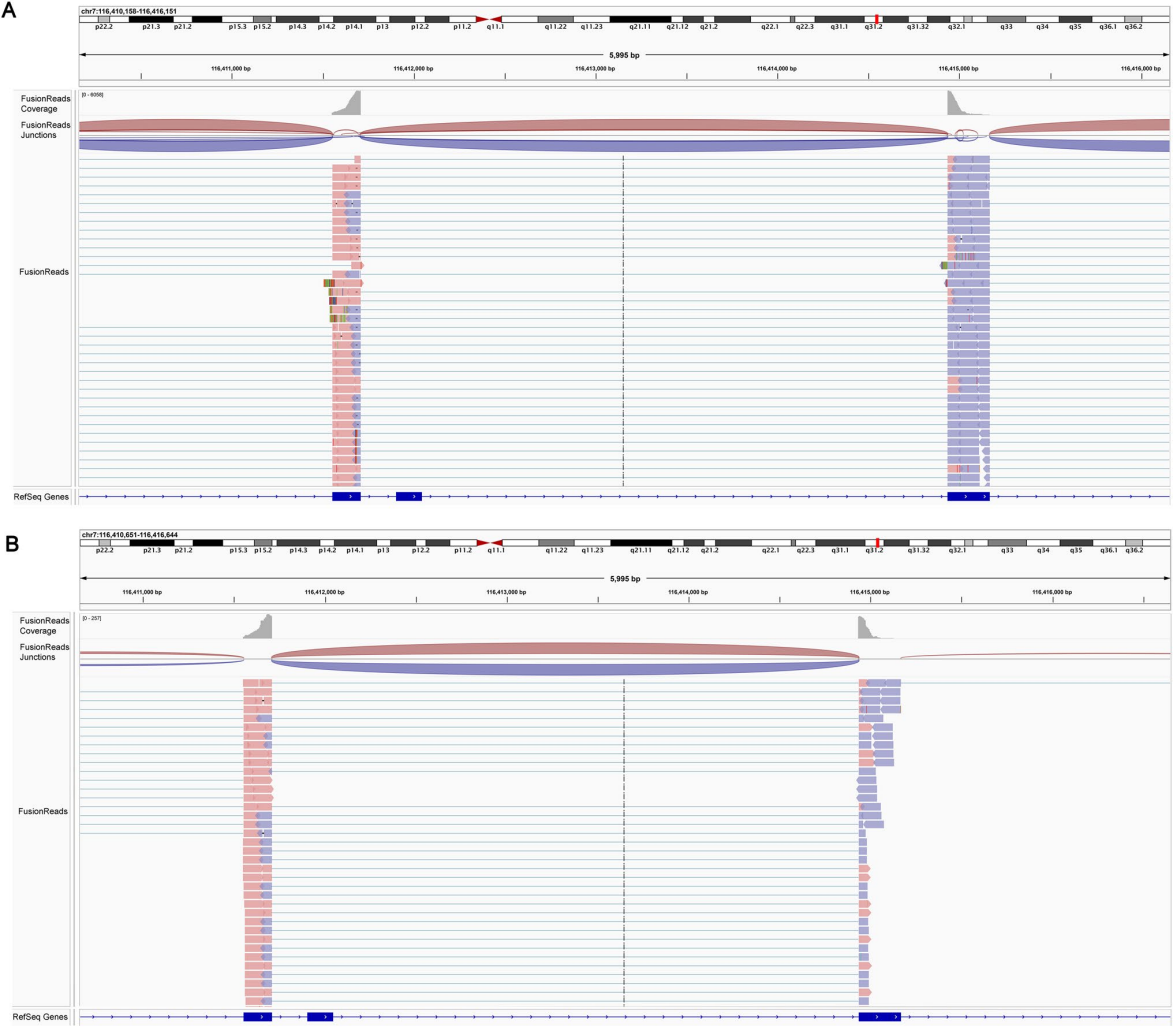
METΔex14 detection using DNA and RNA sequencing. **A** Sequencing of DNA panel showed MET mutation at c.694_695insA (protein: p.S232Yfs*13) and sequencing of RNA panel showed METΔex14 in the patient. **B** Representative METΔex14 data was only detected with sequencing of RNA panel in the patient

In the NSCLC cohort, we found five patients with METΔex14 using DNA or RNA panel sequencing. DNA sequencing showed that 11 NSCLC cases with MET mutation and only one case was MET mutation at a c.3028G > A site, which predicted MET exon 14 skipping in mRNA processing. The METΔex14 was also confirmed using RNA panel sequencing (Fig. 4A). Four cases with METΔex14 were only found through RNA panel sequencing (Fig. 4B). Thus, METΔex14 was easier detected using RNA sequencing compared to DNA sequencing.

### Correlation of SNV/indel/CNV/fusion with clinical characteristics

**Fig. 5:**
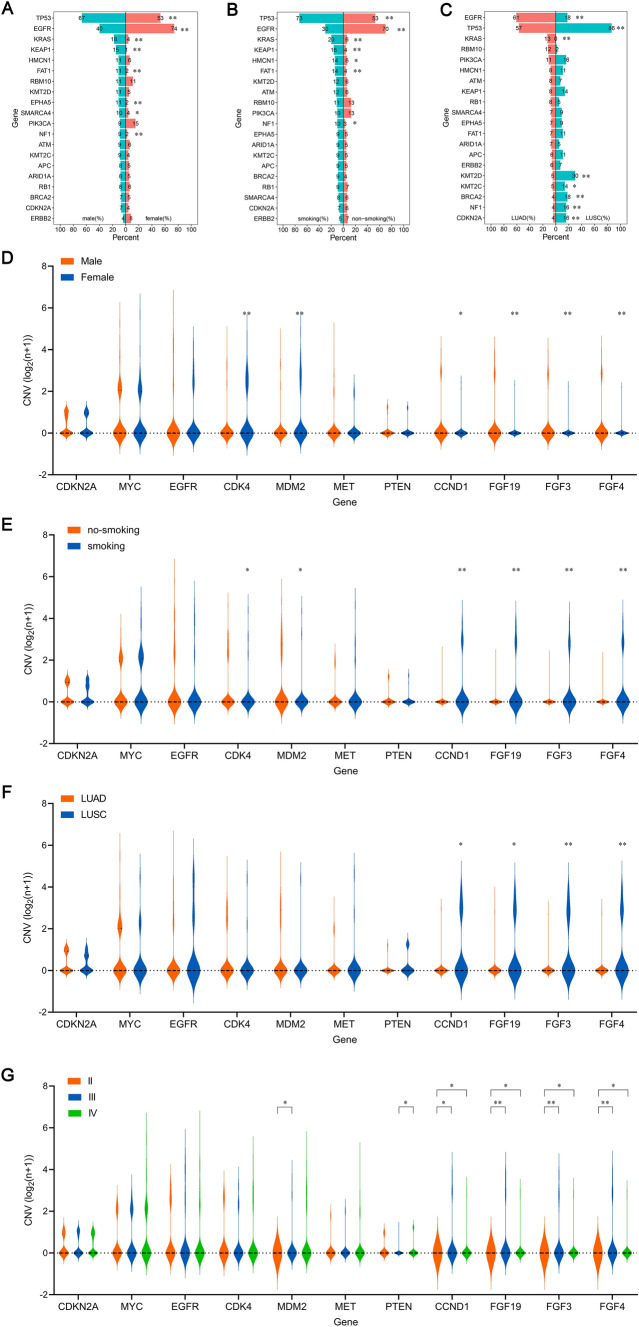
Correlation of gene SNV/indel/CNV/fusion and clinical characteristics in the NSCLC cohort. **A** SNV/indel rates of top 20 genes between male and female in NSCLC cases. Statistics based on the Fisher’s exact test. *p < 0.05, **p < 0.01. **B** SNV/indel rates of top 20 genes between smoking and non-smoking patients with NSCLC. Statistics based on the Fisher’s exact test. *p < 0.05, **p < 0.01. **C** SNV/indel rates of top 20 genes in patients with LUAD or LUSC. Statistics based on the Fisher’s exact test. *p < 0.05, **p < 0.01. **D** CNV difference analysis of NSCLC cases between male and female. Statistics based on the unpaired *t* test. *p < 0.05, **p < 0.01. **E** CNV difference analysis between smoking and non-smoking patients with NSCLC. Statistics based on the unpaired *t* test. *p < 0.05, **p < 0.01. **F** CNV difference analysis in patients with LUAD or LUSC. Statistics based on the unpaired *t* test. *p < 0.05, **p < 0.01. **G** Difference analysis of CNV in NSCLC patients with stage II, III and IV. Statistics based on the One-Way ANOVA test. *p < 0.05, **p < 0.01

We further analyzed correlation of SNV/indel/CNV/fusion with clinical characteristics in the NSCLC cohort. As shown in Fig. 5A–C, SNV/indel rates of TP53, EGFR and KRAS were significantly correlated with gender (Fig. 5A), smoking (Fig. 5B) and cancer subtype (Fig. 5C). Of course, some genes, such as EGFR were significantly different in different age (Figure S1A) and clinical stage (Figure S1B). CNV including cyclin D1 (CCND1) and FGF3/4/19 genes in male, smoking and LUSC was significantly enhanced than no-smoking and LUAD, respectively (Fig. 5D–F). CNV of CCND1 and FGF3/4/19 genes were dramatically different between stage II and III/IV patients (Fig. 5G). But there was no statistical correlation in CNV of many genes with age (Figure S1C). Fusion rates of most genes in different groups according to age (Figure S1D), gender (Figure S1E), smoking (Figure S1F), tumor subtype (Figure S1G) and clinical stage (Figure S1H) exhibited subtle difference in the NSCLC cases. Overall, SNV/indel and CNV of many genes were correlated with some clinicopathological features, such as gender, smoking, cancer subtypes and clinical stages in NSCLC patients.

## Discussion

DNA sequencing for detection of gene fusion has proved to be challenging due to the complexed nature of genomic rearrangements and existence of a variety of intron non-coding regions [13]. Combination of RNA sequencing could comprehensively reveal gene variation features, including SNV/indel, CNV and gene fusion. In the current study, DNA and RNA panels covered all well-documented driver genes, including SNV/indel of EGFR, KRAS, HER2 and BRAF, CNV of MET and HER2, gene fusions of ALK, ROS1, RET and NTRK, and METΔex14, from the CSCO. The rates of EGFR SNV/indel, MET CNV and ALK fusion were 52.1%, 4.1% and 6.0% in 386 NSCLC patients, respectively. DNA panel sequencing accurately revealed somatic SNV/indel and CNV information of tumor genes and RNA panel sequencing was superior in identifying gene fusion compared to DNA panel sequencing. METΔex14 was more easily detected by RNA sequencing, but gene fusion with intergenic region was only detected by DNA sequencing. Thus, RNA sequencing greatly complemented deficiency of DNA sequencing for gene fusion and vice versa, which together provided comprehensive and reliable gene variation features for NSCLC.

RNA sequencing used to detect gene somatic SNV/indel variation is considered to be inaccurate because of high error rate and degeneration of RNA. Only 11% of SNVs with VAF between 5 and 10% in BRAF and RAS genes was detected by whole-transcriptome sequencing compared with WES [11]. Similar findings were reported by another independent study as well [14]. Generally, FISH and IHC are two commonly used methods to detect CNVs in clinical tumor tissue samples. As expedited development of NGS technology in the past decade, DNA NGS has been extensively applied for CNV detection, although there has not been any NGS-based companion diagnostic method approved for CNV detection in NSCLC. So far, there is no consensus on the criteria for determining MET gene amplification with susceptibility or resistance to targeted therapy [15]. In the current study, the expression of EGFR and MET genes were characterized by both parallel DNA and RNA sequencing analysis, which may increase the possibility of identifying appropriate targeted drugs for these patients.

Gene CNV are considered to be potential tumor biomarkers for NSCLC therapy. Apolipoprotein B mRNA editing catalytic polypeptide-like 3B (APOBEC3B) CNV gains were beneficial to immunotherapy response in NSCLC cases [16]. Patients with somatic CNV amplification in myeloid cell leukemia 1 (MCL1) gene exhibited unfavorable OS of NSCLC in a southern Chinese population [17]. CNV of MET and HER2 were recommended by the CSCO guideline for target therapy of NSCLC. In this study, we found that CNV including CCND1, FGF3/4/19 genes in male, smoking and LUSC cases was significantly enhanced than female, no-smoking and LUAD, respectively. Consistently, Heo and the colleagues have reported significant associations between CNV and smoking, such as acyl-CoA thioesterase 1 (ACOT1), N-terminal acetyltransferase F (NAA60), gasdermin-D (GSDMD) and HLA-DPA1 genes [18].

Overall, sequencing of DNA and RNA panels, due to features of fewer tumor samples, more detecting depth and less cost of time and material provided the foundation for wide clinical application in NSCLC. Meanwhile, combination of DNA and RNA panels revealed more gene variations, which might discover potential drug targets and benefit for patients with NSCLC.

## Conclusion

Our findings demonstrated that combination method of DNA and RNA sequencing analysis in detecting multiple types of clinical driver mutations will facilitate the possibility of identifying appropriate targeted drugs for NSCLC patients. More solid conclusion is warranted for further validation of the clinical utility of this combined method in target therapy selection for NSCLC patients in a series of following prospective studies.

### Supplementary Information

**Additional file1:** Supplementary Figure and Supplementary Tables

## Data Availability

Raw data is not publicly available to preserve individuals’ privacy.
