# Supplementary material for: Identification of gene variation feature for targeted therapy of non-small cell lung cancer through combined method of DNA and RNA sequencing
Source: Discov Oncol. 2024 Mar 6;15:67. doi: 10.1007/s12672-024-00915-3 (PMC10917717; doi:10.1007/s12672-024-00915-3)
Supplement: Supplementary file 1 — Additional file1: Supplementary Figure and Supplementary Tables [file 12672_2024_915_MOESM1_ESM.pdf]

Supplementary Materials

Supplementary Figure

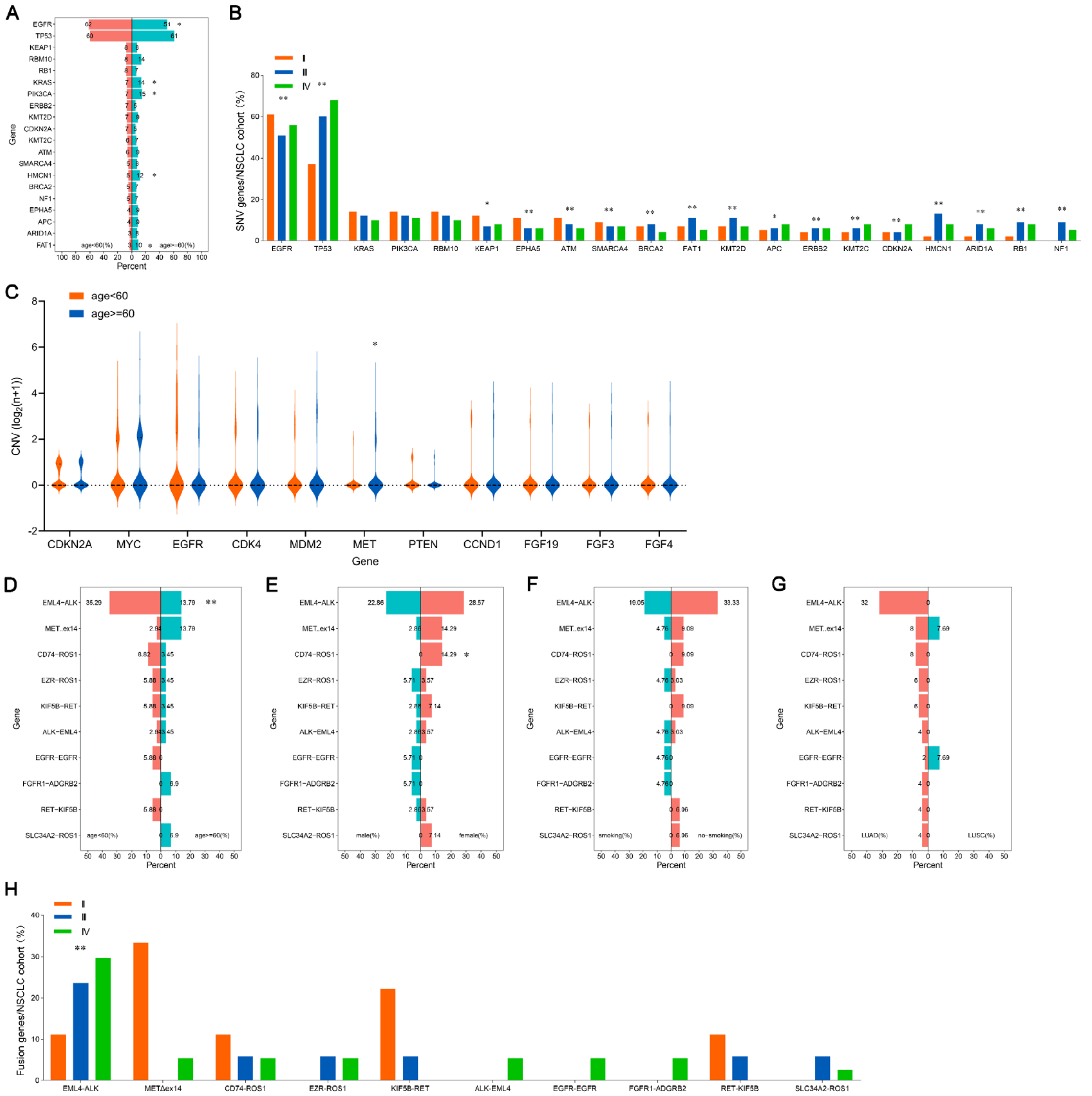

Figure S1. Correlation of gene SNV/indel/CNV/fusion and clinical characteristics in the NSCLC cohort. (A) SNV/indel rates of top 20 genes between age <60 and ≥60 in NSCLC cases. Statistics based on the Fisher's exact test. **p*<0.05. (B) SNV/indel rates of top 20 genes in NSCLC cases with stage II, III and IV. Statistics based on the Fisher's exact test. **p*<0.05, ***p*<0.01. (C) Difference analysis of CNV in NSCLC patients between age <60 and ≥60. Statistics based on the unpaired *t* test. **p*<0.05, ***p*<0.01. (D) Difference analysis of fusion genes between age <60 and ≥60 in NSCLC cases. Statistics based on the Fisher's exact test. ***p*<0.01. (E) Difference analysis of fusion genes in NSCLC cases between male and female. Statistics based on the Fisher's exact test. **p*<0.05. (F) Difference analysis of fusion genes in smoking and non-smoking

patients with NSCLC. Statistics based on the Fisher's exact test. (G) Difference analysis of fusion genes in patients with LUAD or LUSC. Statistics based on the Fisher's exact test. (H) Difference analysis of fusion genes in NSCLC patients with stage II, III and IV. Statistics based on the Fisher's exact test. **p<0.01.

Supplementary Tables

Table S1 769 genes of the DNA panel

ABCA13	ABCA8	ABCB1	ABCC2	ABCC9	ABL1	ACADSB	ACOT13	ACRC(GCNA)	ADCY8
ADGRG6	AGAP1	AK7	AKT1	AKT2	AKT3	ALDH5A1	ALG9	ALK	ALOX12B
ALS2CR11(C2CD6)	AMBRA1	AMER1	ANAPC7	ANKRD28	ANKRD46	ANO1	APAF1	APC	APOL2
APOPT1	AQR	AR	ARAF	ARHGAP26	ARHGAP4	ARHGAP6	ARHGEF12	ARHGEF3	ARID1A
ARID1B	ARID2	ARID4A	ARID5B	ARL13B	ARL4A	ARL6IP6	ARMC5	ASB11	ASH1L
ASPH	ASXL1	ASXL2	ATG3	ATG4C	ATIC	ATM	ATP6V0A1	ATP6V0A2	ATP6V0A4
ATP6V0E1	ATP8A1	ATR	ATRX	AURKA	AURKB	AXIN1	AXIN2	AXL	B2M
BAP1	BARD1	BCAS1	BCL2	BCL2L1	BCL2L11	BCL6	BCOR	BCR	BIRC3
BLM	BMPR1A	BRAF	BRCA1	BRCA2	BRD4	BRIP1	BRMS1L	BRS3	BTF3
BTG1	BTK	C22orf23	C5orf15	C5orf42(CPLANE1)	C7orf66	C8orf34	CAB39	CACNA1E	CACNA2D1
CALD1	CALM2	CALR	CARD11	CASP8	CAST	CBFB	CBL	CBR3	CBR4
CCDC157	CCDC18	CCND1	CCND2	CCND3	CCNE1	CD274	CD40	CD74	CD79A
CD79B	CDA	CDC73	CDCA8	CDH1	CDK12	CDK4	CDK6	CDK8	CDKL3
CDKN1A	CDKN1B	CDKN2A	CDKN2B	CDKN2C	CDO1	CEBPA	CEP120	CEP290	CFAP221
CFAP53	CHD1	CHD2	CHEK1	CHEK2	CHRM3	CIC	CLASP2	CLEC16A	CLEC9A
CNKSR3	CNOT8	COL15A1	COX18	CPS1	CREBBP	CRKL	CRLF2	CSF1R	CSF3R
CTAGE5	CTCF	CTLA4	CTNNB1	CTSC	CUL3	CXCL8	CXCR4	CYBA	CYFIP1
CYLD	CYP19A1	CYP2B6	CYP2C19	CYP2C8	CYP2D6	DARS2	DAXX	DCHS2	DDR1
DDR2	DDX19B	DDX58	DEPDC5	DHFR	DIAPH1	DIAPH2	DICER1	DIS3	DLC1
DMXL1	DNAJB1	DNAJC11	DNMT1	DNMT3A	DNMT3B	DOCK11	DOT1L	DPP6	DPYD
DSCAM	E2F3	EBP	EED	EGFR	EIF1AX	EIF4E	EIF4G3	ELFN1	ELMOD2
EML4	ENOSF1	ENSA	EP300	EPCAM	EPG5	EPHA3	EPHA5	EPHA7	EPHB1
EPYC	ERBB2(HER2)	ERBB3	ERBB4(HER4)	ERCC1	ERCC2	ERCC3	ERCC4	ERG	ERI1
ERRFI1	ESR1	ETV1	ETV4	ETV5	ETV6	EWSR1	EXOSC8	EZH2	EZR
FAM149A	FAM153B	FAM161A	FAM175A	FAM184B	FAM46C(TENT5C)	FANCA	FANCC	FANCD2	FANCF
FANCG	FAS	FAT1	FBXO11	FBXW7	FGF10	FGF16	FGF19	FGF3	FGF4
FGF6	FGFR1	FGFR2	FGFR3	FGFR4	FH	FLCN	FLI1	FLOT1	FLT1(VEGFR1)
FLT3	FLT4(VEGFR3)	FMNL2	FMO1	FMR1	FNBP4	FOLH1B	FOXA1	FOXL2	FOXO1
FOXP1	FUBP1	FUS	FXR1	GABRP	GALNT12	GALNT14	GANC	GATA1	GATA2
GATA3	GIPC1	GLI1	GMEB1	GNA11	GNA13	GNAQ	GNAS	GPAT3	GPC4
GPM6A	GRB10	GREM1	GRIK2	GRIN2A	GSK3B	GSKIP	GSTA1	GSTM1	GSTP1

GUCY1A2	H3F3A	HAUS2	HAUS6	HCAR2	HDGFRP3(HDGFL3)	HERC6	HEY1	HGF	HIST1H1C(H1-2)
HIST1H3B(H3C2)	HLA-A	HLA-B	HLA-C	HMCN1	HNF1A	HNF4A	HOMER1	HRAS	HSD17B11
HSD3B1	HSPA1B	HSPA4	HSPA5	HSPH1	HTT	HYOU1	IARS	ICOSLG	ID2
ID3	IDH1	IDH2	IGF1	IGF1R	IGF2	IKBKE	IKZF1	IL10	IL13RA1
IL7R	IMPG1	INHBA	INPP4A	INPP4B	IRF4	IRF6	IRF8	IRS2	ITGAL
JAK1	JAK2	JAK3	JUN	KDM5A	KDM5C	KDM6A	KDR(VEGFR2)	KEAP1	KIAA1210
KIAA1841	KIF5B	KIT	KLF4	KMT2A	KMT2C	KMT2D	KPNA4	KPNB1	KRAS
KTN1	LAMA3	LATS1	LATS2	LEPR	LMO1	LNPEP	LONRF3	LRP2	LRRC16A(CARMIL1)
LRRC34	LYN	MALRD1	MALT1	MAP2K1(MEK1)	MAP2K2(MEK2)	MAP2K4(MEK4)	MAP3K1	MAP3K13	MAP3K4
MAP4K3	MAP4K5	MAPK1	MAPKAP1	MAPKBP1	MARK1	MARK3	MAX	MCL1	MDC1
MDM2	MDM4	MED12	MED12L	MED14	MED19	MEF2B	MEIS1	MEN1	MET
METTL9	MITF	MLH1	MLH3	MMP16	MMP3	MPL	MRE11A	MRPL19	MS4A13
MSH2	MSH3	MSH6	MTF1	MTF2	MTHFR	MTOR	MTR	MTRR	MUTYH
MYADM	MYB	MYC	MYCL	MYCN	MYD88	MYO10	MYOD1	MYOM1	MZT2A
NAB1	NAB2	NAMPT	NAPG	NAV1	NBAS	NBEAL1	NBN	NCOA6	NCOR1
NEDD4L	NEO1	NF1	NF2	NFE2L2	NFKBIA	NFXL1	NKAP	NKX2-1	NLRP7
NOTCH1	NOTCH2	NOTCH3	NOTCH4	NPM1	NR1I3	NR4A3	NRAS	NRG1	NRG4
NSD1	NT5C2	NTHL1	NTRK1	NTRK2	NTRK3	NUDT13	NUP85	NUP93	OSBP
OTOGL	OTOS	P2RY8	PAK1	PAK7	PALB2	PAPOLG	PAQR8	PARD6B	PARK2(PRKN)
PARP1	PARP2	PARP3	PARP8	PAX3	PAX5	PBRM1	PDCD1	PDCD1LG2	PDE4D
PDGFB	PDGFRA	PDGFRB(PDGFR)	PDPK1	PDS5A	PFKP	PGBD1	PGR	PGRMC2	PHF20
PIGF	PIK3C2G	PIK3C3	PIK3CA	PIK3CB	PIK3CD	PIK3CG	PIK3R1	PIK3R2	PIK3R3
PIM1	PKHD1	PLCG2	PLEKHA1	PLEKHH2	PLXNC1	PMS1	PMS2	PNO1	POLA1
POLD1	POLE	POSTN	PPARG	PPP1R21	PPP2R1A	PRDM1	PRELID3B	PREX2	PRKAR1A
PRKCI	PRKDC	PRPF39	PRPF4	PTCH1	PTEN	PTK2	PTPN11	PTPN4	PTPRD
PTPRJ	PTPRS	PTPRT	PURA	RAB2B	RABGAP1L	RAC1	RAD21	RAD50	RAD51
RAD51B	RAD51C	RAD51D	RAD52	RAD54L	RAF1	RALGAPB	RAP2B	RARA	RASA1
RB1	RBM10	RBM27	RECQL4	REL	RET	RFC1	RFWD2(COP1)	RHOA	RHOT1
RIC1	RICTOR	RIPK2	RIT1	RNF112	RNF19A	RNF43	ROBO1	ROS1	RPF2
RPRD1A	RPS6KB1	RPTOR	RRM1	RRP1B	RUNX1	RWDD1	RYBP	RYR2	SASH1

SCOC	SDHA	SDHAF2	SDHB	SDHC	SDHD	SEL1L3	SEMA3C	SEMA3E	SERTAD4
SETD2	SF3B1	SFXN4	SH2D1A	SHQ1	SHROOM3	SIMC1	SIPA1L2	SKA3	SLC13A1
SLC22A2	SLC25A13	SLC30A5	SLC31A1	SLC34A2	SLC35B1	SLC7A8	SLC9C2	SLCO1B1	SLCO1B3
SLIT1	SLX4	SMAD2	SMAD3	SMAD4	SMARCA4	SMARCB1	SMO	SNX6	SOCS1
SOD2	SOX17	SOX2	SOX9	SPEN	SPOP	SRC	SRSF3	SRY	SS18
STAB2	STAG2	STARD4	STAT3	STK11(LKB1)	STMN1	STRBP	STT3A	STYX	SUCLG1
SUFU	SUGCT	SUZ12	SYK	SYNE2	TAF15	TAOK3	TARBP1	TBC1D8B	TBCD
TBX3	TECPR2	TENM3	TERT	TET1	TET2	TFDP1	TFRC	TGFBR1	TGFBR2
TMEM126B	TMEM127	TMEM132D	TMEM67	TMPRSS15	TMPRSS2	TMTC4	TNFAIP3	TNFRSF14	TNFSF13B
TNIK	TNKS	TNRC18	TOP1	TOP2B	TP53	TP63	TPH1	TPM1	TRA2A
TRAF7	TRIM24	TRIM25	TSC1	TSC2	TSHR	TSN	TTC1	TTC6	TTN
TUBD1	TXNDC16	TXNRD1	U2AF1	UBAP2L	UBE2E3	UBE4A	UBN2	UBXN7	UGT1A1
ULK2	ULK4	UMPS	UPF2	USP11	USP34	USP9Y	UTS2	UTY	VEGFA
VHL	VSIG10	WDR5	WHSC1(NSD2)	WHSC1L1(NSD3)	WT1	XIAP	XPC	XPO1	XRCC1
XRCC2	YAP1	YLPM1	YWHAE	ZBBX	ZBTB40	ZDHHC17	ZDHHC20	ZMYM2	ZMYM4
ZNF195	ZNF2	ZNF280D	ZNF283	ZNF367	ZNF711	ZNF805	ZNF91	ZZZ3	

Table S2 29 genes of the RNA panel

ALK	BRAF	CD74	EGFR	EML4	ERG	ESR1	ETV1	ETV4	ETV5
ETV6	EWSR1	FGFR1	FGFR2	FGFR3	FGFR4	KIF5B	MET	NRG1	NTRK1
NTRK2	NTRK3	PDGFRA	PDGFRB（PDGFR）	RET	ROS1	SLC34A2	SLC45A3	TPM3	
